# Mevalonate pathway activity as a determinant of radiation sensitivity in head and neck cancer

**DOI:** 10.1002/1878-0261.12535

**Published:** 2019-07-26

**Authors:** Natalia Ricco, Amy Flor, Don Wolfgeher, Elena V. Efimova, Aishwarya Ramamurthy, Oliver K. Appelbe, Jacqueline Brinkman, Andrew W. Truman, Michael T. Spiotto, Stephen J. Kron

**Affiliations:** ^1^ Department of Molecular Genetics and Cell Biology The University of Chicago IL USA; ^2^ Department of Biological Sciences University of North Carolina at Charlotte NC USA; ^3^ Department of Radiation and Cellular Oncology University of Chicago Medical Center IL USA; ^4^ Department of Radiation Oncology University of Illinois Hospital and Health Sciences System Chicago IL USA

**Keywords:** DNA damage response, HNSCC, mevalonate pathway, radiation sensitivity, senescence, statin

## Abstract

Radioresistance is a major hurdle in the treatment of head and neck squamous cell carcinoma (HNSCC). Here, we report that concomitant treatment of HNSCCs with radiotherapy and mevalonate pathway inhibitors (statins) may overcome resistance. Proteomic profiling and comparison of radioresistant to radiosensitive HNSCCs revealed differential regulation of the mevalonate biosynthetic pathway. Consistent with this finding, inhibition of the mevalonate pathway by pitavastatin sensitized radioresistant SQ20B cells to ionizing radiation and reduced their clonogenic potential. Overall, this study reinforces the view that the mevalonate pathway is a promising therapeutic target in radioresistant HNSCCs.

**Abbreviations**
a.u.: arbitrary units
DSB: double‐strand break
FDPS: farnesyl diphosphate synthase
FPP: farnesyl pyrophosphate
GGPP: geranylgeranyl pyrophosphate
GGPS: geranylgeranyl diphosphate synthase
HMGCR: 3‐hydroxy‐methylglutaryl HMG‐CoA reductase
HMGCS1: 3‐hydroxy‐3‐methylglutaryl‐CoA synthase 1
HNSCC: head and neck squamous cell cancer
IPP: isopentenyl diphosphate
IR: ionizing radiation
LC‐MS/MS: liquid chromatography tandem mass spectrometry
LDL: low‐density lipoprotein
LDLR: low‐density lipoprotein receptor
LFQ: label‐free quantification
LRP1: prolow‐density lipoprotein receptor‐related protein 1
M5P: mevalonate‐5‐phosphate
M5PP: mevalonate‐5‐diphosphate
MFI: mean fluorescence intensity
MVD: mevalonate decarboxylase
MVK: mevalonate kinase
NSDHL: NAD(P) dependent steroid dehydrogenase‐like
PIT: pitavastatin
PMVK: phosphomevalonate kinase
SD: standard deviation
STR: short tandem repeats

## 1 Introduction

Radiotherapy remains one of the most widely used treatments for cancer as nearly half of patients receive it alone or in combination with other types of therapies (Delaney *et al*., 2005). For some tumor types, evidence indicates that dose escalation can improve the efficacy of radiotherapy (Andrews *et al*., 2004) (Bartelink *et al*., 2007) (Kuban *et al*., 2008), but the consequence is an increase in normal tissue toxicity, which has been documented as a major limitation of chemoradiation therapy (Eisbruch *et al*., 2002).

Head and neck squamous cell carcinomas include a broad category of neoplasms that primarily develop in the oral cavity, pharynx, and larynx and are the sixth leading cause of cancer worldwide with approximately half a million new cases every year (Boyle and Levin, 2008) (Hunter *et al*., 2005). In the past decade, the survival rate for HNSCC has not significantly changed, with two‐thirds of HNSCC patients presenting with locally advanced disease and five‐year survival rates remaining below 50% (Carvalho *et al*., 2005). Radical radiotherapy in combination with platinum‐based chemotherapy remains the standard treatment for HNSCC with locoregionally advanced carcinoma (J. Pignon *et al*., 2000). Common acute toxicities include dysphagia, mucositis, dermatitis, and dysgeusia (altered taste sensation), with xerostomia (reduced salivary output) the most common and most adversely affecting quality of life in patients (Bjordal *et al*., 2001). Other cytotoxic cancer agents including targeted drugs can be combined with radiotherapy (Bonner *et al*., 2006) (J.‐P. Pignon *et al*., 2009), though toxicities may be dose‐limiting and resistance is common and associated with a poor prognosis.

An attractive alternative strategy is to identify agents that are nontoxic and likely to be ineffective on their own but can serve as radiosensitizers (Dickreuter *et al*., 2016) (Khan *et al*., 2010) (Leiker *et al*., 2015) (Su *et al*., 2013), potentially allowing treatment of patients with safer radiation doses. An ideal outcome would be increasing the efficacy of radiation on the tumor while at the same time decreasing its adverse effects on normal tissue. One promising class of drugs is the statins, a family of chemically related competitive inhibitors of 3‐hydroxy‐3‐methylglutaryl‐coenzyme A reductase (HMGCR), the first committed enzyme of the mevalonate pathway (Hristov *et al*., 2009) (Stancu and Sima, 2001) (Alagona, 2010). As the mevalonate pathway serves a key role in cholesterol biosynthesis, statins are commonly used to treat dyslipidemia and to prevent cardiovascular disease. Statins lead to an overall decrease in circulating low‐density lipoprotein (LDL) cholesterol both by inhibiting cholesterol biosynthesis and increasing cholesterol uptake via compensatory upregulation of the LDL receptor. Their broad use and high overall safety have permitted large‐scale studies to identify additional impacts on patients, revealing potential benefits in cancer prevention and treatment. Retrospective studies to identify factors affecting success of radiotherapy for prostate cancer have found that incidental use of statins, as for treatment of dyslipidemia, confers improved responses (Hutchinson and Marignol, 2017). Along with extensive preclinical studies, the clinical data suggest that statins may display cancer selectivity, increasing radiosensitization in tumor cells while protecting normal tissue from late radiation effects.

In earlier work, we identified several statin drugs as candidate radiosensitizers in a screen for ionizing radiation‐induced foci (IRIF), a proxy for DNA double‐strand break (DSB) repair (Labay *et al*., 2011). Following up on this screen, we tested effects of several statins on irradiated mammary and melanoma tumor cells, finding that lipophilic statins delayed DNA repair and promoted therapy‐induced senescence *in vitro* and *in vivo* (Efimova *et al*., 2017). These results suggested examining the mevalonate pathway as a target to enhance radiation sensitivity in HNSCC, a malignancy where radiation resistance is a common clinical challenge. This stimulated a hypothesis‐driven proteomics analysis comparing two radioresistant HNSCC cell lines, JSQ3 and SQ20B (Weichselbaum *et al*., 1986, 1988), to a radiosensitive control, SCC61 (Weichselbaum *et al*., 1986), to explore contributions of the mevalonate pathway to the proteomic signature of radioresistance. Indeed, radioresistance was not only associated with dysregulated mevalonate pathway activity but also increased sensitivity to statins with respect to proliferation, viability, DNA damage response, and radiation sensitization. We found that cholesterol levels were increased in radioresistant cells, along with elevated LDL receptor abundance and uptake of extracellular LDL. Further, consistent with these *in vitro* results, in patients treated for HNSCC with radiotherapy, incidental use of statins was associated with improved local control of tumors. These studies suggest potential benefit to concomitantly prescribing lipophilic statin drugs along with radiation therapy in order to improve outcomes for head and neck cancers.

## 2 Materials and methods

### 2.1 Cell lines, cell culture, and agents

Head and neck squamous cell carcinoma cell lines SCC61 (radiosensitive) derived from a glossal tumor (Weichselbaum *et al*., 1986), JSQ3 (radioresistant) derived from a nasal vestibule tumor, and SQ20B (radioresistant) derived from a laryngeal tumor (Weichselbaum *et al*., 1988) were grown in DMEM/F‐12 media (Life Technologies, Grand Island, NY, USA) supplemented with 20% fetal bovine serum (Denville Scientific) and penicillin/streptomycin (100 units·mL^−1^, 100 mg·mL^−1^, Life Technologies). Cultures were maintained at 37 °C in a humidified atmosphere containing 5% CO_2_ until approximately 70% confluence was reached. Authenticity was confirmed by short tandem repeats (STR) profiling (Center for Genomic Research, University of Illinois at Chicago). All the cell lines tested negative for mycoplasma contamination.

Pitavastatin calcium (PIT) was obtained from Atomole. Simvastatin, pravastatin, atorvastatin, lovastatin, and rosuvastatin were from the NIH Clinical Collection (BioFocus, Mechelen, Belgium). FTI (FTI‐276) and GGTI (GGTI‐2147) inhibitors were from CalBiochem.

Cellular irradiation was delivered using a ^60^Co gamma irradiator (GammaCell, MDS Nordion) with a dose rate ranging from 9.4 to 7.6 cGy·s^−1^, depending on the date of the experiment.

### 2.2 LC‐MS/MS proteomics

#### 2.2.1 Sample preparation

SCC61, JSQ3, and SQ20B were seeded at 10^6^ cells in 100 mm Petri dishes and were grown under standard conditions for 48 h. Proteins were isolated with M‐PER (Thermo Scientific, Fitchburg, WI, USA) in the presence of protease and phosphatase inhibitors (Thermo Scientific). Thirty microgram of the extract was separated by SDS/PAGE electrophoresis on a 12% MOPS‐buffered gel (Thermo Scientific) run for 10 min at 200V resulting in a ~2‐cm ‘gel plug’. After staining with Imperial Protein Stain (Thermo Scientific), individual samples were excised by razor blade and chopped into ~1‐mm^3^ pieces. Each sample was washed in distilled H_2_O and destained using 100 mm NH_4_HCO_3_ pH 7.5 in 50% acetonitrile. A reduction step was performed by addition of 100 μL 50 mm NH_4_HCO_3_ pH 7.5 and 10 μL of 200 mm Tris (2‐carboxyethyl) phosphine HCl at 37 °C for 30 min. The proteins were alkylated by addition of 100 μL of 50 mm iodoacetamide prepared fresh in 50 mm NH_4_HCO_3_ pH 7.5 buffer and allowed to react in the dark at 20 °C for 30 min. The gel fragments were washed in water, then acetonitrile, and vacuum‐dried. In‐gel trypsin digestion was carried out overnight at 37 °C with 1 : 50–1 : 100 enzyme–protein ratio of sequencing grade‐modified trypsin (Promega, Fitchburg, WI, USA) in 50 mm NH_4_HCO_3_ pH 7.5, and 20 mm CaCl_2_. Peptides were extracted first with 5% formic acid, then with 75% ACN: 5% formic acid, combined, and vacuum‐dried.

#### 2.2.2 High‐pressure liquid chromatography (HPLC)

All samples were re‐suspended in Burdick & Jackson HPLC‐grade water containing 0.2% formic acid (Fluka, Muskegon, Michigan, USA), 0.1% TFA (Pierce, Rockford, IL, USA), and 0.002% Zwittergent 3–16 (Calbiochem, San Diego, CA, USA), a sulfobetaine detergent that contributes the following distinct peaks at the end of chromatograms: MH^+^ at 392, and in‐source dimer [2 M + H^+^] at 783, and some minor impurities of Zwittergent 3–12 seen as MH^+^ at 336. The peptide samples were loaded onto a 0.25 μL C_8_ OptiPak trapping cartridge custom‐packed with Michrom Magic (Optimize Technologies, Oregon City, OR, USA) C8, washed, then switched in‐line with a 20 cm by 75 μm C_18_ packed spray tip nanocolumn packed with Michrom Magic C18AQ, for a 2‐step gradient. Mobile phase A was water/acetonitrile/formic acid (98/2/0.2), and mobile phase B was acetonitrile/isopropanol/water/formic acid (80/10/10/0.2). Using a flow rate of 350 nL·min^−1^, a 90 min, 2‐step LC gradient was run from 5% B to 50% B in 60 min, followed by 50–95% B over the next 10 min, 10 min at 95% B, then back to the starting conditions and re‐equilibrated.

#### 2.2.3 LC‐MS/MS analysis

The samples were analyzed via electrospray tandem mass spectrometry (LC‐MS/MS) on a Thermo Q‐Exactive Orbitrap mass spectrometer, using a 70 000 RP survey scan in profile mode, m/z 360–2000 Da, with lockmasses, followed by 20 MS/MS HCD fragmentation scans at 17,500 resolution on doubly and triply charged precursors. Singly charged ions were excluded, and ions selected for MS/MS were placed on an exclusion list for 60 s.

Label‐free quantification (LFQ) sample sets were analyzed as described previously (Truman *et al*., 2012). All LC‐MS/MS *.raw data files were analyzed with maxquant version 1.5.2.8, searching against the SPROT Human database (Downloaded: 1/07/2016 with isoforms) using the following criteria: LFQ was selected for quantitation with a min of 1 high confidence peptide to assign LFQ intensities. Trypsin was selected as the protease with max miss cleavage set to 2. Carbamidomethyl(C) was selected as a fixed modification. Variable modifications were set to Oxidization (M), Formylation (n‐term), Acetyl (Protein n‐term), Deamidation (NQ), GlyGly (K), HNE (Budach *et al*., 2005), and Phosphorylation (STY). Orbitrap mass spectrometer was selected using an MS error of 20 ppm and a MS/MS error of 0.5 Da. 1% FDR cutoff was selected for peptide, protein, and site identifications.

Ratios were reported based on the LFQ intensities of protein peak areas determined by MaxQuant and reported in the proteinGroups.txt. Proteins were removed from this results file if they were flagged by MaxQuant as ‘Contaminants’, ‘Reverse’, or ‘Only identified by site’. Three complete biological replicates were performed. LFQ peak intensities were analyzed in each run to determine protein hits that fell into the category of single‐condition‐only hits and retained if they maintained this state across all runs. Proteins with two out of three observations were retained and quantitated based on LFQ intensity. Log_2_ transformation of ratios was performed, and a significance cutoff of 1.5‐fold change was implemented (±0.58 log_2_ ratio). Gene Ontology analysis was performed by submitting significant hits (≥log_2_ 0.58 and ≤ log_2_ −0.58) and those only seen in either radioresistant (SQ20B or JSQ3) or radiosensitive (SCC61) lists to a statistical overrepresentation test using bioinformatics software (Panther 14.0) (The Gene Ontology Consortium, 2016) (Mi *et al*., 2013) (Muruganujan *et al*., 2016). Significant gene lists for (SQ20B/SCC61) and (JSQ3/SCC61) were compared to the *Homo sapiens* reference list using Fisher's exact test for determining *P* value and a Benjamini–Hochberg procedure to calculate false discovery rate (FDR). Results were sorted by fold‐enrichment for GO_BP (biological pathway), GO_MF (molecular function), GO_CC (cell component), and Reactome Pathway annotations results.

### 2.3 Western blotting

About 1 × 10^6^ cells were seeded in 10‐cm‐diameter culture dishes, incubated overnight, harvested, and pelleted by centrifugation at 1000 ***g***. Total cell lysates were prepared using ice‐cold RIPA buffer (Thermo Fisher) in the presence of protease inhibitors (Halt protein inhibitor cocktail, Thermo Fisher) including EDTA (Thermo Fisher). Protein concentration was determined by BCA assay (Thermo Fisher). About 20 μg of protein was loaded per well, separated using a 12% Bis‐Tris gel (Life Technologies), and electroblotted onto PVDF membrane (Bio‐Rad, Hercules, CA, USA). Equal loading was verified by actin detection (1:10 000 Sigma‐Aldrich, St. Louis, Missouri, USA, A‐5316). Immunoblotting was performed using a rabbit monoclonal primary antibody targeting HMGCR (1:1000, Abcam, Cambridge, UK, ab174830) followed by anti‐rabbit peroxidase‐conjugated secondary antibody (GE) followed by detection with chemiluminescent peroxidase substrate (ECL Femto, Thermo Fisher). Blot imaging was conducted using an iBright FL1000 Imaging System (Life Technologies).

For HMGCS1 and NSDHL 0.5 × 10^6^ cells were plated in 10‐cm‐diameter culture dishes, and after the indicated treatments, total cell lysates were prepared using M‐PER cell lysis buffer (Thermo) in the presence of protease and phosphatase inhibitors (Thermo). About 20–30 μg of protein was loaded per well, separated using SDS/PAGE gels (Bio‐Rad), and electroblotted onto a PVDF membrane (Millipore, Burlington, MA, USA). Equal loading was verified by actin detection (Sigma‐Aldrich). Immunoblotting for targets was performed using primary rabbit, mouse, or goat anti‐human antibodies and species‐specific peroxidase‐conjugated secondary antibodies (Millipore, Jackson Laboratories) followed by detection with ECL Femto chemiluminescent peroxidase substrate (Thermo). Antibodies: HMGCS1 (1:200, Santa Cruz, sc‐32422), NSDHL (1:2000, Abcam, ab190353), Actin (1:10,000, Sigma‐Aldrich, A‐5316).

### 2.4 Cholesterol measurement

Total cholesterol content from 0.7 × 10^6^ cells was measured per sample with a colorimetric assay kit (Cell Biolabs, Inc). Measurements were performed for three biological replicates per group. Cells were lysed in chloroform:isopropanol:NP‐40 (7:11:0.1) for 30 seconds using a mini bead beater (BioSpec). Homogenized cells were centrifuged at 15,000 × g for 10 min. The resulting organic phase layer was air‐dried at 50 °C, and the remaining organic solvent was removed by using a vacuum concentrator (Eppendorf) for 30 min. The lipid pellets were resuspended in the 1X assay diluent included in the kit. Following the kit protocol, relative cholesterol concentrations in the samples were determined by absorbance at 570 nm using a microplate reader (Synergy Neo, BioTek).

### 2.5 Colony formation assay

200 cells per well were plated in 6‐well plates the day before treatment. Pitavastatin was added at the indicated doses 1 h prior to irradiation. Two weeks later, cells were fixed and stained with 0.1% crystal violet (Sigma‐Aldrich) for 5 to 10 min. Wells were rinsed with water, dried, and imaged with a Nikon D70s camera. For quantitation plots, the number of cell colonies with greater than 50 cells was counted manually in each well‬.

### 2.6 Cell viability assays

For the ATP luminescence cell viability assay, 10,000 cells per well were plated in 96‐well white polystyrene plates (Thermo) in a final volume of 100 μL. 24 h after plating, cells were treated with different pitavastatin concentrations and some plates were exposed to different radiation doses. 48 h after treatment, an ATP‐based cell viability assay (Cell Titer‐Glo Kit, Promega) was used to measure the relative amount of ATP by luminescence following manufacturer's instructions. Luminescence was measured with a microplate reader (Synergy Neo, BioTek). Three biological replicates were measured.

For the flow cytometry‐based Calcein Violet viability assay, cells were plated overnight in 6‐well culture plates at 0.2 x 10^6^ cells per well in complete culture medium as described above. The following day, cells were treated with pitavastatin (10 μm) for 1 h followed by irradiation (0 or 10 Gy). Cells were then returned to a 37 °C, 5% CO_2_ environment for 48 h prior to cell viability assay, conducted as follows. Cells were harvested by cell scraper into the medium contained in each culture well. The cell suspensions were transferred to sterile tubes, and cells were collected by centrifugation for 5 min at 1200 x g. Cell pellets were resuspended in Calcein Violet 450 AM viability stain (1 μm, eBiosciences) in 1% BSA‐DPBS for 20 min on ice in the dark. Flow cytometry analysis was then conducted using a Fortessa flow cytometer (BD) equipped with a 405‐nm excitation laser and a 450/50‐nm emission detector. Fluorescence data for 10,000 cells were acquired per sample. Cellular viability data were analyzed using FlowJo software (FlowJo).

### 2.7 LDL receptor immunostaining and LDL uptake assays

HNSCC cells were plated at 0.2 × 10^6^ cells per well in 6‐well plates for 24 h in complete culture medium. For LDL receptor staining, cells were harvested by cell scraper to preserve cell surface receptors, counted, and resuspended in 0.5% BSA‐DPBS at 0.5 × 10^6^ cells per sample for staining. 0.5% BSA‐DPBS served as the blocking agent for 10 min on ice. A fluorescently labeled antibody targeting LDLR (Abcam clone EP1553Y, Alexa Fluor 647 conjugate) was then added to cells at the titration recommended by manufacturer for 30 min on ice. Sytox Blue viability stain was added at 1 μm for 10 min on ice just prior to flow cytometry analysis. Two biological replicates were analyzed per cell line.

For LDL uptake assays, an LDL uptake assay kit was used according to manufacturer specifications (Cayman Chemical, Ann Arbor, MI, USA). Briefly, cells were plated at 0.1 × 10^6^ cells per well in 6‐well plates for 48 h in complete culture medium. At 48 h, serum‐free medium was exchanged on all samples to provide a lipid‐free growth environment, to which fluorescent LDL‐DyLight 488 (Cayman Chemical) was added at manufacturer recommended dilution (1 : 200). Samples for microscopy were incubated for 6 h, washed 2X with kit assay buffer, and imaged using a fluorescent microscope with an appropriate fluorescence filter set (Zeiss). A set of biological replicates for flow cytometry were incubated for 6 h, harvested by cell scraper, pelleted by centrifugation for 5 min at 1,200 x g, and resuspended in kit assay buffer (Cayman Chemical). 7‐AAD viability stain was added for 10 min on ice just prior to flow cytometry analysis.

Flow cytometry was conducted using a Fortessa cytometer (Becton Dickinson, Franklin Lakes, NJ, USA) equipped with excitation lasers and emission detectors appropriate for the fluorophores used in the experiment. Data for 10,000 cells per sample were acquired using FACSDiva software. Postacquisition analysis was conducted using FlowJo software (FlowJo, LLC).

### 2.8 Neutral comet assay

To analyze DSB, cells were plated at 0.2 x 10^6^ cells per well in 6‐well plates the day before treatment. PIT was administered at the indicated doses 1 h prior to 10 Gy irradiation. After 20 h, cells were mixed with Comet LM agarose (Bio‐Rad) and single‐cell electrophoresis was performed on CometSlides (Trevigen). Slides were fixed, dried, stained with SYBR Green DNA stain (1/10 000×; Life Technologies), and imaged on an Axiovert 40 with a 20X Plan‐NeoFluar objective and an AxioCam monochromatic fluorescence camera. Images were analyzed using a Comet assay macro for ImageJ software (http://www.med.unc.edu/microscopy/resources/imagejplugins-and-macros/comet-assay).

### 2.9 Senescence‐associated β‐galactosidase (SA‐β‐Gal) assay

0.5 x 10^6^ cells were plated in a 100‐mm dish the day before treatment. Pitavastatin (10 μm) was administered 1 h prior to 10 Gy irradiation. After 6 days, cells were washed with PBS, harvested by scraping, and resuspended in 1 ml of culture medium. Cells were incubated with Bafilomycin A1 (1 μm, Thermo) for 30 min at 37 °C with rotation to neutralize lysosomal pH. DDAO galactoside (10 μg·mL^−1^) was added, and cells were incubated for 1 h at 37 °C with rotation. After centrifugation at 4 °C for 5 min at 1200 x g, cells were resuspended in ice‐cold 1% BSA‐DPBS and centrifuged again. Supernatant was discarded, and cells were resuspended in serum‐free media with 1 μm Calcein Violet 450 AM (eBioscience) for 15 min on ice. Stained cells were with examined with a Fortessa flow cytometer and analyzed with FlowJo to measure DDAO to detect SA‐β‐Gal and calcein violet to identify viable cells.

### 2.10 Patient study population and analysis

We utilized a retrospective head and neck cancer database of 803 patients to identify patients with Stage III‐IVB disease treated for curative intent at the University of Illinois Medical Center at Chicago between 1990 and 2012. Patients treated with medications other than a statin or having Stage I‐II disease were excluded resulting in 518 patients for analysis. Analysis was performed under University of Illinois Medical Center IRB protocol 2011‐1075 in accordance with the ethical standards of the responsible committee on human experimentation and with the Helsinki Declaration of 1999, as revised in 2000. Data were collected from all available physical and electronic medical records. Time to local control (LC) or distant control (RC) was determined from last date of radiotherapy. Patterns of failure were determined as the first failure with any component of local, regional, or distant recurrence, respectively.

### 2.11 Statistical analysis

For cell‐based assays, statistical significance was determined using the unpaired t‐test (cholesterol assays), unpaired multiple t‐test (ATP viability assays), or Mann–Whitney U‐test (Comet assays and flow cytometry). Most experiments were performed in biological duplicate or triplicate. Flow cytometry experiments comprised *n* > 3000 viable cells per sample. *P* value ≤ 0.05 was considered statistically significant. Calculations were performed using Prism software (GraphPad).

For patient data analysis, discrete variables were compared with the Chi‐square test and differences in the medians were assessed using the Wilcoxon rank sum test. We used JMP version 10 (SAS Institute) to perform statistical analysis using two‐sided tests and defining significance as *P* < 0.05. For univariate and multivariate analyses, we used Cox proportional hazard models to compare differences in survival. Censoring is assumed to be noninformative. Variables with *P* value < 0.1 on univariate analysis were included on multivariate analysis. Survival curves were plotted using the Kaplan–Meier method, and significance was assessed using the Log Rank test.

### 2.12 Cholesterol uptake assay

For cholesterol uptake assays, an assay kit was used according to manufacturer's specifications (Cayman Chemical). Briefly, cells were plated at 0.2 x 10^6^ cells per well in 6‐well plates for 24 h in complete culture medium. At 24 h, serum‐free medium was exchanged on all samples to provide a lipid‐free growth environment. NBD cholesterol was then added to the serum‐free media at the recommended dilution (20 μg·mL^−1^). At this point, inhibitors were added, including PIT (2.5 μm) and U‐18666A (1/1000X manufacturer stock solution, Cayman Chemical). Cells were incubated for 24 h, harvested by cell scraper, pelleted by centrifugation for 5 min at 1200 x g, and resuspended in kit assay buffer (Cayman Chemical) for flow cytometry analysis.

Flow cytometry was conducted using a Fortessa cytometer (Becton Dickinson) equipped with excitation lasers and emission detectors appropriate for the fluorophores used in the experiment. Data for 10,000 cells per sample were acquired using FACSDiva software. Postacquisition analysis was conducted using FlowJo software (FlowJo, LLC).

## 3 Results

### 3.1 Proteomic profiling of radioresistant HNSCCs

**Figure 1:**
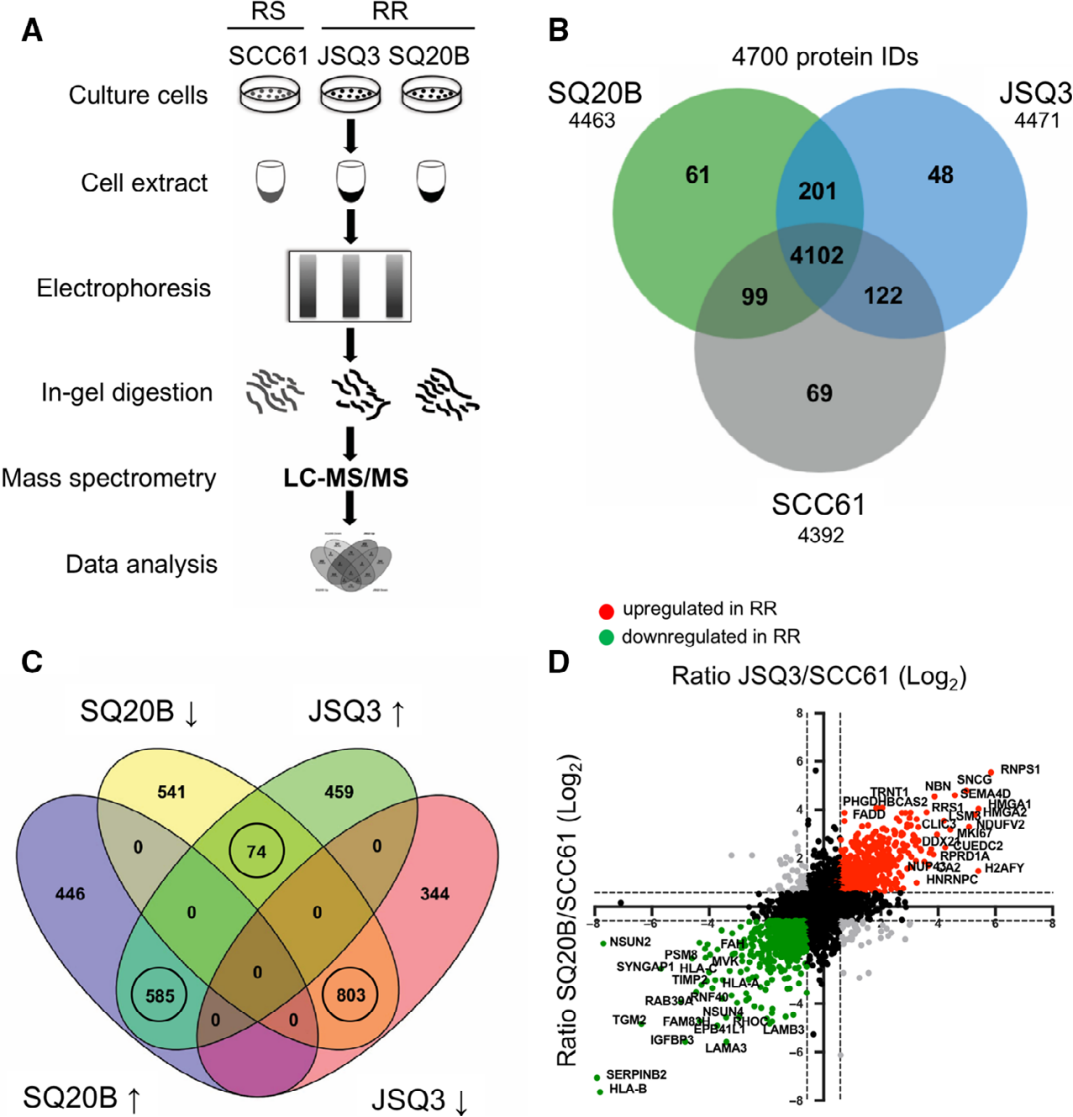
Proteomic analysis reveals a distinct radioresistant HNSCC cell proteome. (A) Protocol schematic for mass spectrometry assay. Whole‐cell protein lysates were prepared from SCC61 (radiosensitive), JSQ3 (radioresistant), and SQ20B (radioresistant) HNSCC cell lines. Proteins were separated by gel electrophoresis, digested with trypsin, and analyzed via LC‐MS/MS. (B) Venn diagram showing proteins uniquely identified in one or two cell lines, or proteins shared between all cell lines. 201 proteins were shared by the two radioresistant cell lines (SQ20B and JSQ3) and 69 proteins were unique to radiosensitive cells (SCC61), while 4102 proteins were shared by all three cell lines. (C) Venn diagram showing distribution of up‐ and downregulated proteins shared between the two radioresistant cell lines, SQ20B and JSQ3, compared to the radiosensitive cell line, SCC61. In general, most proteins found in both radioresistant cell lines were similarly regulated, with 585 shared proteins upregulated and 803 shared proteins downregulated. Only 74 proteins displayed variable regulation. Only circled groups were considered for further analysis. (D) Representation of quantitative mass spectrophotometric intensity ratios of SQ20B/SCC61 (*x*‐axis) and JSQ3/SCC61 (*y*‐axis) shown on log_2_ scale. A 1.5‐fold (0.58 log_2_) change was considered statistically significant. Upregulated proteins, red. Downregulated proteins, green. Variably regulated proteins, gray; nonsignificant proteins, black. The data show a linear trend, with proteins up‐ or down‐regulated in the SQ20B vs. SCC61 cell line tending to be similarly regulated in JSQ3 vs. SCC61 samples.

We set out to uncover molecular determinants of radiation resistance in HNSCCs by performing proteome profiling (Fig. 1A). Radiosensitive (SCC61) and radioresistant (JSQ3 and SQ20B) HNSCC human cancer cell lines were grown under standard culture conditions, and after protein isolation and digestion in biological triplicates, peptides were analyzed by LC‐MS/MS mass spectrometry. Using MaxQuant software and accepting only protein identifications with a minimum FDR of 1%, a total of 4700 different proteins were identified, of which 4392 were present in the SCC61 samples, 4471 in JSQ3, and 4463 in SQ20B (Fig. 1B).

In order to analyze the proteomic signature of radioresistant cells, label‐free quantitation (LFQ) was conducted using the mass spectrometry intensities for all the protein hits identified in a minimum of 2 of 3 biological replicates. Radioresistant LFQ values for each protein ID were compared to those of the radiosensitive cell line. A 1.5‐fold (0.58 log_2_) change was considered statistically significant. We observed a total of 585 proteins significantly upregulated and 803 significantly downregulated in each of the radioresistant cell lines compared with the radiosensitive cell line (Fig. 1C). Individual proteins in these categories were then further evaluated (Fig. 1D). Proteins strongly upregulated in the radioresistant cell lines included RNPS1, SNCG, SEMA4D, HMGA1, HMGA2, and several others. Downregulated proteins in radioresistant cell lines included HLA‐B, SERPINB2, TGM2, IGFBP3, LAMA3, and others. The full proteomic data set was uploaded to the ProteomeXchange repository with identifier PXD007188. A supplementary data file including all significant protein hits and GO pathways was uploaded to the https://figshare.com/ under https://doi.org/10.6084/m9.figshare.8145971.

### 3.2 Proteomics analysis suggests a role for dysregulated mevalonate pathway activity in HNSCC radioresistance

**Figure 2:**
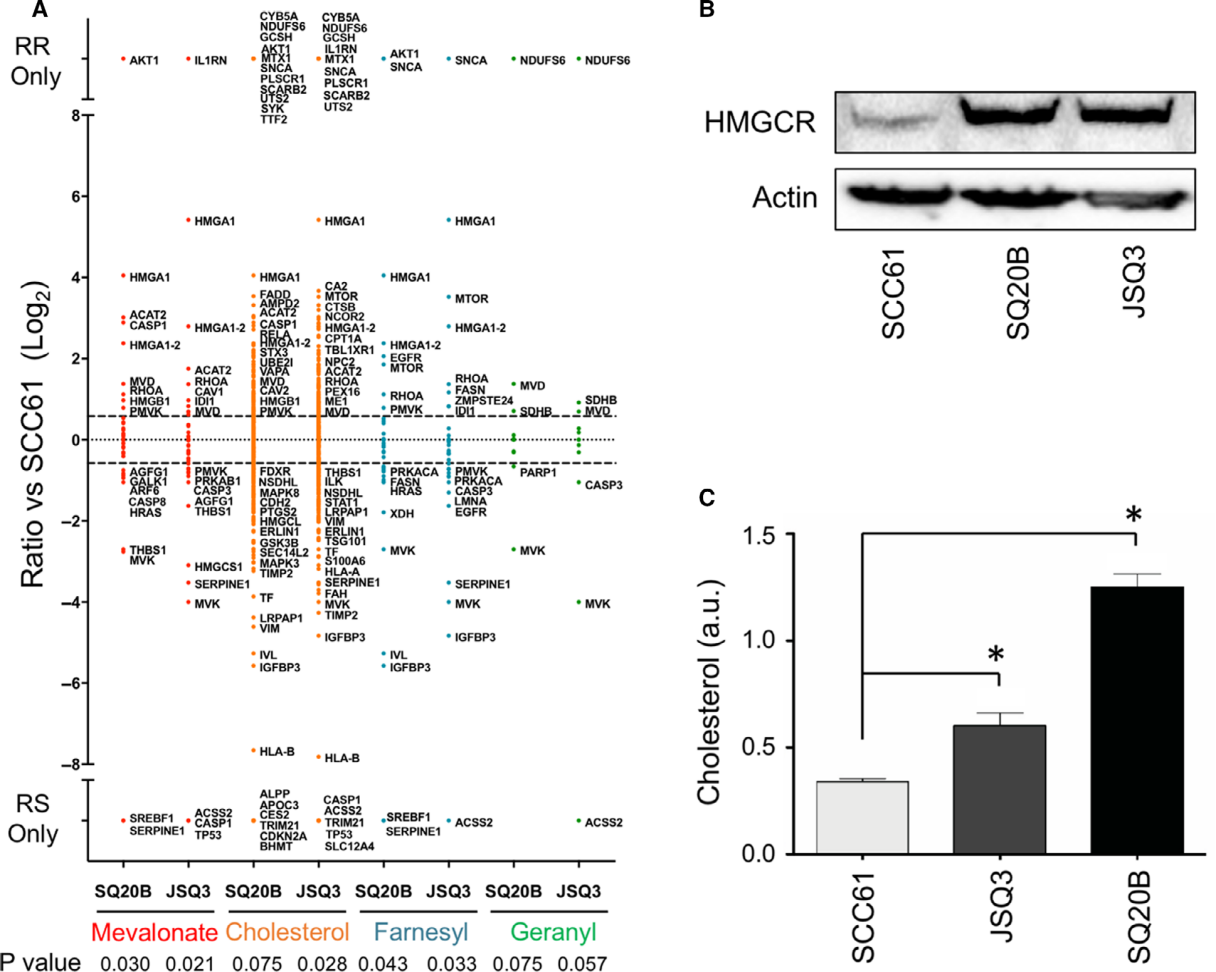
Gene ontology analysis of proteomic data of mevalonate pathway activity and substrate validation. (A) Proteins were separated into functional categories by gene ontology (GO) terms related to mevalonate pathway activity, including mevalonate biosynthetic pathway, cholesterol biosynthesis, protein farnesylation, and protein geranylgeranylation. Protein data are shown as the log_2_ fold‐change ratio for SQ20B/SCC61 (labeled SQ20B) and JSQ3/SCC61 (labeled JSQ3). (B) Amount of HMGCR protein is increased in radioresistant cells. Cell samples were collected during active growth, lysed, and a western blot was performed. (C) Mean cholesterol levels in SCC61, JSQ3, and SQ20B 48 h after cell passage (during active growth). 0.7 × 10^6^ cells were assayed per sample. Statistical significance was determined using the parametric unpaired t‐test. Error bars: SD,* n* = 3 biological replicates. Significance is indicated by asterisks (*P < 0.05). Cholesterol levels were significantly increased in the radioresistant cell lines.

To determine a proteomic ‘signature’ of radioresistant cells and evaluate potential links to mevalonate pathway activity, we first performed gene ontology (GO) analysis on the hit list of proteins that showed significant variability between radioresistant and radiosensitive cell lines. We then searched for GO terms of relevance to our hypothesis. Metabolism‐related GO terms of significance included glucose metabolic process (GO0006006, *P* = 2.06 × 10^−2^ and 5.12 × 10^−3^), protein metabolic process (GO0019538, *P* = 4.84 × 10^−17^ and 3.13 × 10^−19^), and lipid metabolic process (GO0006629, *P* = 1.73 × 10^−2^ and 9.31 × 10^−5^) (Fig. S1). We also evaluated the significance of GO terms specifically related to mevalonate biosynthesis and metabolism (Fig. 2A), including mevalonate pathway (GO0019287, *P* = 0.030 for SQ20B/SCC61 and 0.021 for JSQ3/SCC61), cholesterol biosynthetic process (GO0006695, *P* = 0.075 and 0.028), protein farnesylation (GO0018343, *P* = 0.043 and 0.033), and protein geranylgeranylation (GO0018344, *P* = 0.075, and 0.057).

Expression profiles of individual protein hits identified to be differentially regulated in the mevalonate pathway (Fig. 2A) suggest dysregulation of this pathway in radioresistant HNSCC. According to LFQ intensities, HMGCS1, the initiator enzyme of the pathway, was significantly downregulated in the JSQ3 radioresistant cells. We confirmed the downregulation of HMGCS1 in both radioresistant cell lines by western blotting (Fig. S2). Following the conversion of acetoacetyl‐CoA to HMG‐CoA by HMGCS1, the enyzme HMGCR converts HMG‐CoA to mevalonate. HMGCR is a key rate‐limiting enzyme in the mevalonate pathway. HMGCR was not detected by LC‐MS/MS analysis, but western blotting revealed the enzyme to be upregulated in both radioresistant cell lines (Fig. 2B). Downstream from HMGCR are the enzymes MVK, PMVK, and MVD, which function to convert mevalonate to several intermediate metabolites (mevalonate‐5‐phosphate, M5P and mevalonate‐5‐diphosphate, M5PP) resulting in the formation of isopentenyl diphosphate (IPP). Of these enzymes, LC‐MS/MS analysis indicated that MVK was downregulated, PMVK was variably regulated, and MVD was upregulated in radioresistant cell lines. For enzymes required for conversion of IPP to geranyl pyrophosphate (GGPS), and then to farnesyl pyrophosphate (FDPS), differences across cell lines were insignificant. The enzyme NSDHL, which mediates the conversion of farnesyl pyrophosphate to cholesterol, was found to be downregulated in both radioresistant cell lines. NSDHL downregulation was confirmed by western blotting (Fig. S2). Taken together, these results implicate dysregulation of mevalonate pathway protein expression in radioresistant HNSCC.

An important metabolic outcome of mevalonate pathway activity is cholesterol biosynthesis. Suggesting a functional consequence of mevalonate pathway dysregulation on cell cholesterol content, analysis of cholesterol in untreated cell lysates (Fig. 2C) revealed significantly higher levels of cholesterol in the radioresistant cell lines (*P* < 0.05 by unpaired *t*‐test). These data raised the question whether cholesterol biosynthesis might offer a target to restore radiation sensitivity in radioresistant HNSCC.

### 3.3 Lipophilic statins inhibit cell proliferation in radioresistant HNSCC cell lines

**Figure 3:**
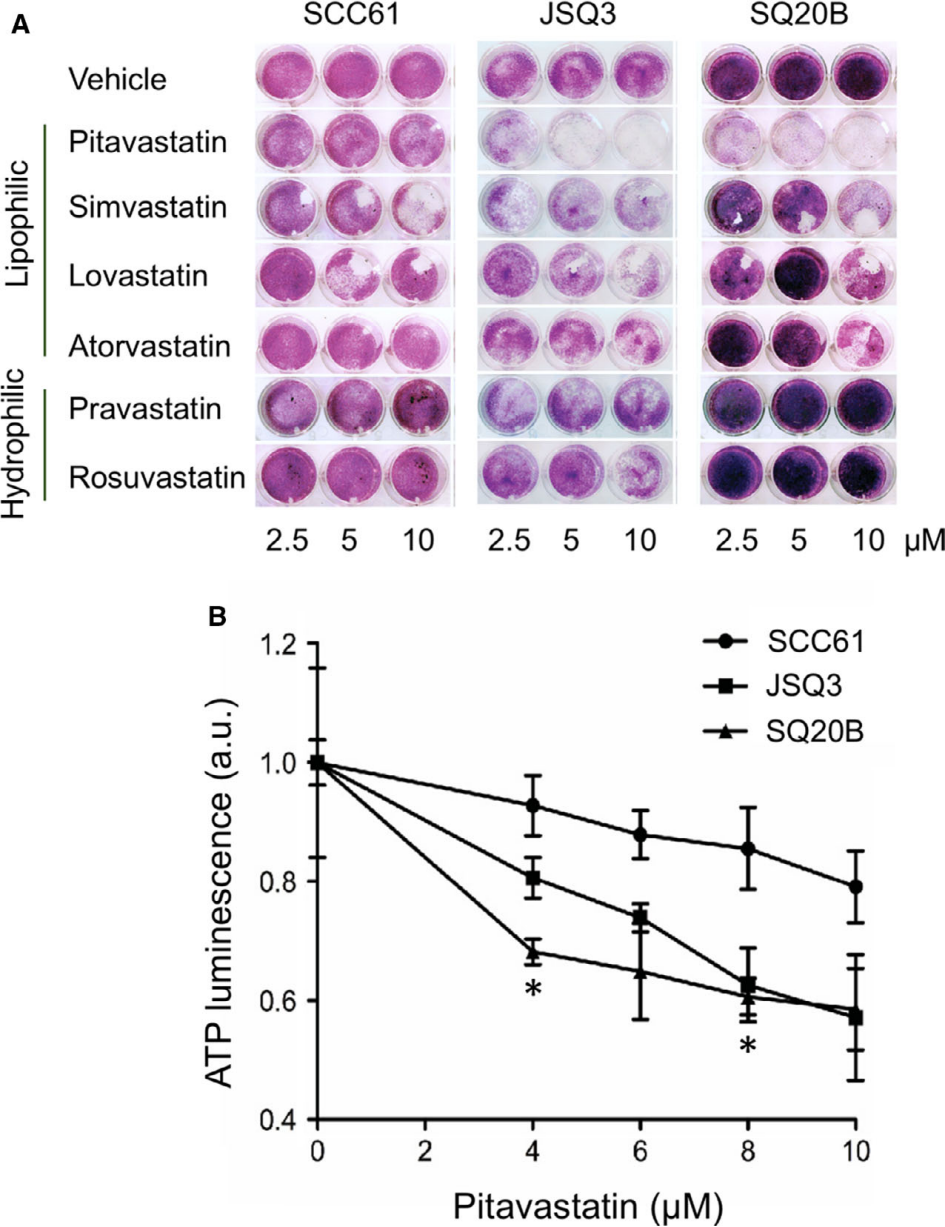
Inhibition of mevalonate pathway by pitavastatin inhibits cellular proliferation and cholesterol levels. (A) Colony formation assay of SCC61, SQ20B, and JSQ3 treated with statins. SCC61, SQ20B, and JSQ3 cells were treated with 2.5, 5, or 10 μm pitavastatin, simvastatin, lovastatin, atorvastatin (lipophilic), or pravastatin or rosuvastatin (hydrophilic) for 1 h prior to IR. After 4 days of culture, crystal violet staining was performed and plates were imaged. Lipophilic statins, notably pitavastatin, and reduced colony formation in cell lines. (B) ATP luminescence viability assay was performed on SCC61, JSQ3, and SQ20B cells to measure viability 48 h after exposing cells to 0, 4, 6, 8, or 10 μm PIT. Three biological replicates were measured; mean ATP luminescence and standard deviation are shown. Viability was reduced by PIT in all cell lines, but markedly so in the radioresistant cell lines, particularly SQ20B. Statistical significance was determined using the parametric unpaired multiple t‐test. Significant conditions are indicated by asterisks (*P < 0.05).

To ascertain the role of the mevalonate pathway in survival of HNSCC cell lines, we tested a panel of FDA‐approved statin drugs, which block mevalonate generation. Statins included lipophilic pitavastatin (PIT), simvastatin, lovastatin, and atorvastatin, as well as the hydrophilic statins pravastatin and rosuvastatin. Using a colony formation assay (Fig. 3A), we evaluated whether any statin might suppress cellular proliferation in HNSCC cell lines. Indeed, growth of cells was suppressed by PIT at doses as low as 2.5 μm, as well as the other lipophilic statins simvastatin, lovastatin, and atorvastatin at higher doses (10 μm). These effects were particularly dramatic for the radioresistant cell lines. The hydrophilic statins pravastatin and rosuvastatin displayed only minimal effects. Given these results, PIT was selected as the most promising statin for further testing in the study.

Results of an ATP‐based cell viability assay (Fig. 3B) further demonstrate that radioresistant cell lines have increased sensitivity to PIT (0 to 10 μm), as measured after 48 hours of treatment. While PIT treatment reduced viability in all cell lines, its effects were more dramatic on radioresistant cell lines. At 4 and 8 μm, PIT treatment significantly reduced viability in SQ20B cells compared with the same doses used on SCC61 cells (*P* < 0.05 by unpaired multiple *t*‐test, *n* = 3 biological replicates per sample). At 10 μm, PIT reduced viability in both radioresistant cell lines to approximately 50% of radiosensitive cells.

### 3.4 Cholesterol uptake via the LDL receptor is elevated in radioresistant HNSCC cell lines

**Figure 4:**
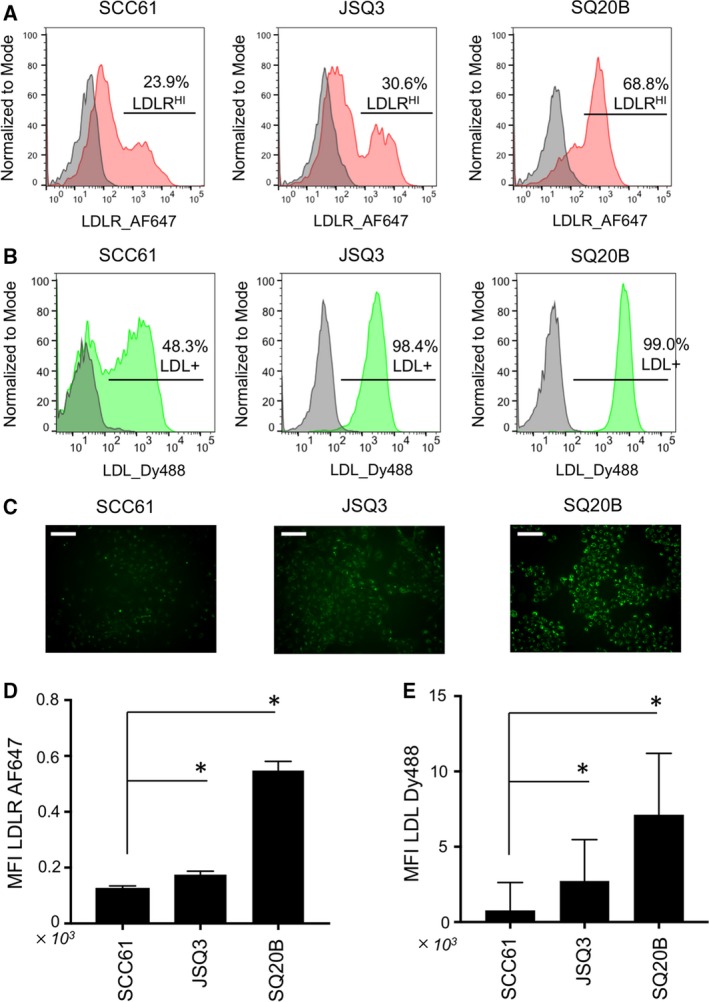
Elevation of LDL receptor levels and LDL uptake characterize radioresistant HNSCC cells. (A) Immunostaining of LDL receptor, LDLR, on living HNSCC cell lines analyzed by flow cytometry. (B) Uptake of fluorescently labeled LDL‐DyLight 488 by HNSCC cells analyzed by flow cytometry. (C) Microscopy images of biological replicates of cell populations treated as shown in (B); scale bars, 20 μm. Elevated levels of LDL receptors and LDL uptake are evident in the radioresistant cell lines JSQ3 and SQ20B. (D) Statistical analysis of LDLR staining intensity. (E) Statistical analysis of LDL‐Dy488 uptake intensity. Mean fluorescence intensities are shown; error bars = SEM. Significance is indicated by asterisks (*P < 0.05). Statistical significance of E and D was determined using Mann–Whitney *U*‐test.

Cellular cholesterol uptake is commonly mediated by endocytosis of low‐density lipoprotein (LDL) through the LDL receptor. Thus, we immunostained for cell surface LDL receptors (Fig. 4A) and examined uptake of fluorescently labeled LDL (Fig. 4B,C). Flow cytometry analysis revealed that levels of LDL receptors were upregulated (LDLR^HI^) on the surface of radioresistant (JSQ3, 30.6%; SQ20B, 68.8%) vs. (SCC61, 23.9%) HNSCC cells. In turn, the radioresistant cell lines demonstrated higher levels of LDL uptake (JSQ3, 98.4%; SQ20B, 99.0%; vs. SCC61, 48.3%). Differential uptake of fluorescently labeled LDL can also be seen in microscopy images taken with standardized exposure and equal cell density across cell lines (Fig. 4C). Statistical analysis of mean staining intensities for LDLR (Fig. 4D) and LDL‐Dy488 uptake (Fig. 4E) confirmed significant upregulation of these features in the radioresistant cell lines (*P* < 0.05 by Mann–Whitney *U*‐test, *n* > 3000 viable cells per sample).

### 3.5 Inhibition of mevalonate pathway sensitizes radioresistant HNSCC cell lines to radiation

**Figure 5:**
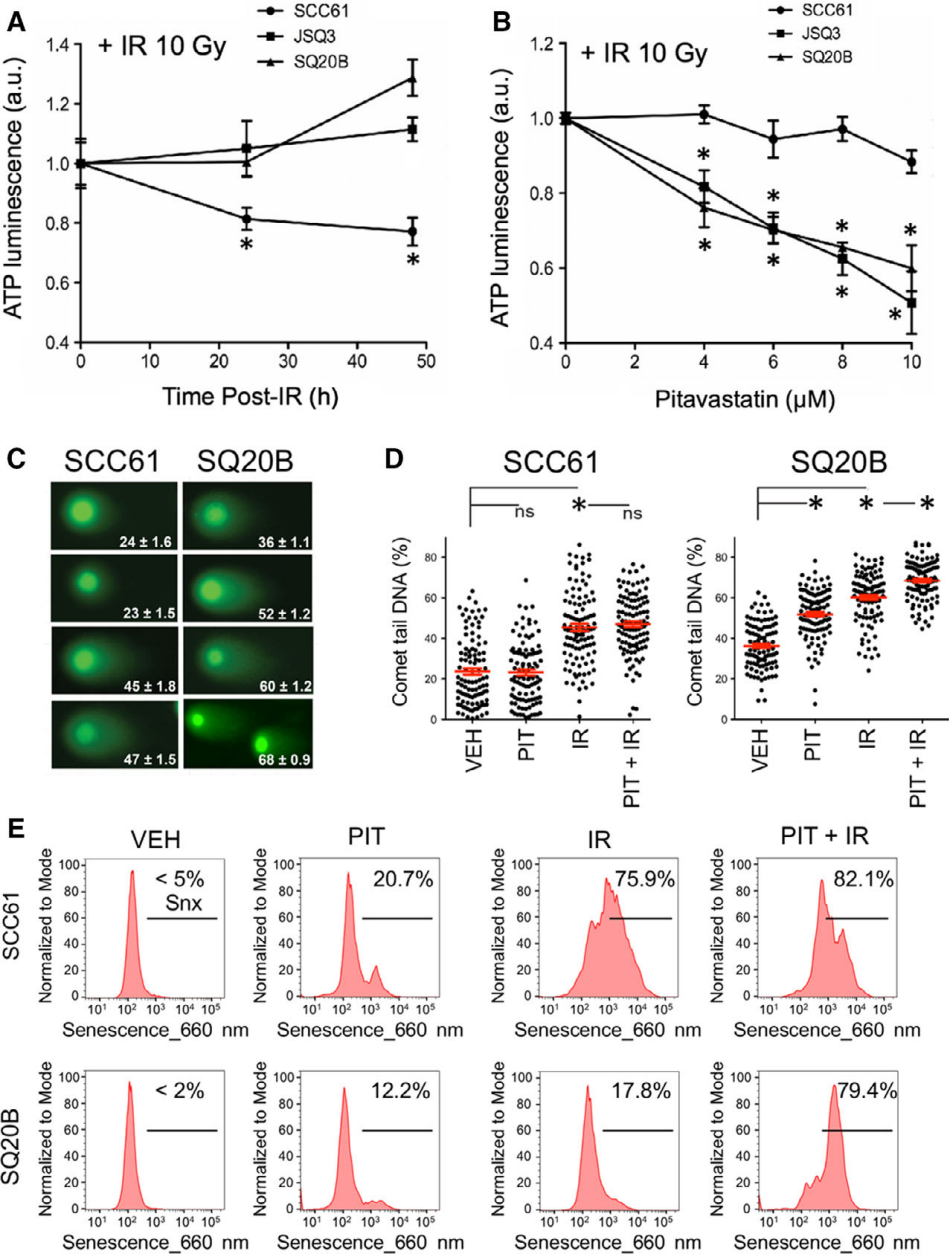
Inhibition of mevalonate pathway by pitavastatin reduces viability, increases DNA damage, and induces senescence upon ionizing radiation in radioresistant HNSCC. (A) ATP‐based cellular proliferation assay of cell lines treated with IR (10 Gy) to demonstrate cell line radiosensitivity. Radioresistant cell lines JSQ3 and SQ20B exhibited lower ATP levels at 48 hr than SCC61 (radiosensitive) cells, which continued proliferating normally (*P < 0.05 vs radiosensitive cells at same time point) Statistical significance was determined using the parametric unpaired multiple t‐test. (B) ATP‐based proliferation assay for cell lines treated with IR (10 Gy) ± PIT (0–10 μm). Radioresistant cell lines demonstrated significantly reduced ATP levels with PIT treatment (*P < 0.05 vs radiosensitive cells at same treatment condition). Statistical significance was determined using the parametric unpaired multiple *t*‐test. (C) Representative images of ‘Comet’ DNA double‐strand break (DSB) assay data with mean percent of DNA in comet tail ± SEM shown. (D) Plots representing mean percent of DNA in comet tail ± SEM for 100 cells per treatment condition. Significance is indicated by asterisks (*P < 0.05). Statistical significance was determined using Mann–Whitney U‐test. Results indicate that PIT enhances persistent DNA DSBs induced by IR in radioresistant SQ20B cells. (E) Flow cytometric senescence assay data of cells treated with 10 μm of PIT alone (PIT), IR only (10 Gy), or in combination with IR (PIT + IR). Vehicle‐treated cells (VEH) were stained as controls. Cells were analyzed by flow cytometry 6 days after treatment. The combination of PIT + IR dramatically elevated the percent of senescent cells above IR alone in the SQ20B radioresistant cells.

To demonstrate the differences in radioresponse across cell lines, an ATP‐based cell viability assay was performed. After a single 10 Gy dose of IR, the radioresistant cell lines JSQ3 and SQ20B maintained viability according to cellular ATP assay for at least 50 h after irradiation (Fig. 5A), while the radiosensitive cell line SCC61 displayed a significant loss of viability over the same period (*P* < 0.05 by unpaired multiple *t*‐test, *n* = 3 biological replicates per sample). The ATP assay further demonstrated the radiosensitizing effects of PIT at concentrations from 1 to 10 μm (Fig. 5B), particularly in radioresistant cells. At 10 μm, PIT reduced ATP in irradiated cells by 15% (SCC61), 40% (SQ20B), or 50% (JSQ3). All dose conditions of PIT tested significantly reduced ATP levels in both radioresistant cell lines as compared to the same dose in the radiosensitive cell line (*P* < 0.05 by unpaired multiple *t*‐test, *n* = 3 biological replicates per sample).

In prior studies of PIT as a radiosensitizer (Efimova *et al*., 2017), we observed that when MCF7 mammary carcinoma cells were treated with PIT and then irradiated, they displayed a marked delay in double‐strand break (DSB) repair. To examine whether PIT similarly affects DSB repair in HNSCC cells, we performed neutral comet assays after treatment with PIT and/or ionizing radiation (IR) (Fig. 5C,D). As expected, the combination of PIT + IR (68 ± 0.9% comet tail DNA) significantly increased the levels of persistent DSBs over that of IR alone (60 ± 1.2%) in SQ20B cells (*P* < 0.05 by Mann–Whitney *U*‐test). This effect was not seen in the sensitive cell line SCC61 (47 ± 1.5% for PIT + IR vs. 45 ± 1.8% for IR only). Further, even without radiation, PIT increased the basal level of DSBs in SQ20B cells. These effects were recapitulated in JSQ3 cells (Fig. S3A). These effects may underlie the loss of proliferation and viability induced by PIT in the radioresistant cell lines.

Along with increasing persistence of DNA damage, our prior work showed that combining PIT with radiation also promoted therapy‐induced senescence in melanoma and breast cancer cells (Efimova *et al*., 2017). Here, senescence of HNSCC cells treated with PIT with or without IR was measured by a conventional flow cytometry SA‐β‐Gal assay at six days after treatment (Fig. 5E). While treating radioresistant SQ20B cells with PIT or IR alone slightly increased senescence (12.2% for PIT alone, 17.8% for IR alone, compared to < 2% in vehicle only treated SQ20B cells), combining statin with radiation induced senescence in 79.4% of cells. A similar pattern was observed in radioresistant JSQ3 cells (Fig. S3B). The senescence response in radiosensitive SCC61 cells was comparable for combination‐treated cells above levels induced by IR treatment alone (75.9 vs 82.1%). Statistical analysis of mean senescence probe fluorescence (Fig. S3C) supports these conclusions (*P* < 0.05 for most conditions compared with controls, Mann–Whitney U‐test, *n* > 3000 viable cells per sample).

To examine effects of treatment with various statins on radioresponse, SCC61, JSQ3, and SQ20B cell lines were treated with statins before irradiation (IR) and assayed for colony formation (Fig. S4). PIT alone decreased colony formation in radioresistant cells. The lipophilic statins simvastatin, lovastatin, and atorvastatin potentiated the effect of radiation on SQ20B cells. The hydrophilic statins tested failed to confer toxicity or radiosensitization in any cell line.

### 3.6 Clinically approved lipophilic statin drugs are associated with better local tumor control in HNSCC patients

Radiotherapy is widely used in the treatment of head and neck cancers, and our previous results suggest that its combination with statins may have beneficial effects. Consequently, we analyzed 518 HNSCC patients on Stage III–IV treated with radiotherapy of which 53 patients were also taking with a statin drug.

Table 1 describes patient characteristics. Patients receiving statin were significantly older, had higher comorbidity scores but less alcohol use, and fewer T3–T4 tumors and N2‐3 nodal disease. Of the 53 patients incidentally taking a statin drug during their radiotherapy, 41 (77.4%) were taking a lipophilic statin and 3 (5.7%) were taking a hydrophilic statin. For 9 patients, the type of statin was not indicated. Table 2 shows univariate and multivariate analysis of survival. On univariate analysis, improved survival was associated with statin use (HR 0.47, 95% CI, 0.20–0.93; *P* = 0.03). On multivariate analysis, statin use remained associated with improved survival (HR 0.29, 95% CI, 0.09–0.71; *P* = 0.004).

**Table 1:** Patient characteristics (*n *= 518).

	Statin (*n* = 53)	No statin (*n* = 465)	*P* value
Median follow‐up (months)	18.4 (11.4–38.7)	18.7 (6.8–58.7)	0.87
Age			
≤57	14 (26.4%)	258 (56.0%)	**<0.0001**
>57	39 (73.6%)	203 (44.0%)	
Gender			
Male	41 (77.4%)	350 (75.3%)	0.74
Female	12 (22.6%)	115 (24.7%)	
Comorbidity			
Medium	16 (30.2%)	366 (78.7%)	**<0.0001**
High‐very high	37 (69.8%)	99 (21.2%)	
Performance status			
<70	5 (9.4%)	22 (4.7%)	0.31
≥70	40 (75.5%)	380 (81.7%)	
Not stated	8 (15.1%)	63 (13.6%)	
Alcohol use			
≥3 drinks/day	26 (63.4%)	276 (76.7%)	0.06
<3 drinks/day	15 (36.6%)	84 (23.3%)	
Smoking			
≥10 pack‐year	39 (81.2%)	350 (79.2%)	0.74
<10 pack‐year	9 (18.8%)	92 (20.8%)	
T‐stage			
T1–2	21 (39.6%)	119 (25.6%)	**0.04**
T3–4	32 (60.4%)	346 (74.4%)	
N‐stage			
N0–1	31 (58.5%)	199 (42.8%)	**0.03**
N2–3	22 (41.5%)	266 (57.2%)	
Primary site			
Oral cavity	15 (28.3%)	116 (25.0%)	0.36
Oropharynx	18 (34.0%)	119 (25.6%)	
Larynx	12 (22.6%)	97 (20.9%)	
Hypopharynx	3 (5.7%)	48 (10.3%)	
Nasopharynx	1 (1.9%)	27 (5.8%)	
Other	4 (7.6%)	58 (12.5%)	
Induction chemotherapy			
Yes	9 (17.0%)	161 (34.6%)	**0.006**
No	44 (83.0%)	304 (65.4%)	
Concurrent chemotherapy			
Yes	47 (88.7%)	313 (67.3%)	**0.0005**
No	6 (11.3%)	152 (32.7%)	
Post‐operative radiotherapy			
Yes	18 (34.0%)	180 (38.7%)	0.50
No	35 (66.0%)	285 (61.3%)	
Type of statin			
Lipophilic	41 (77.4%)	N.D.	
Hydrophilic	3 (5.7%)	N.D.	
Not stated	9 (17.0%)	N.D.	

Included in propensity matching: age, gender, year of diagnosis, facility, path stage grouping, path T‐stage, path N‐stage, LN > 2, LVSI, LND and site. For patient data analysis (table 1) discrete variables were compared with the Chi‐square test and differences in the medians were assessed using the Wilcoxon Rank Sum test, significance was defined as P < 0.05. In patients under statin treatment, variables such as age, comorbidity, T and N‐stage and succes in induction and concurrent chemotherapy, were significantly changed compared to those who where not in treatment.

**Table 2:** Univariate and multivariate analysis of survival (*n* = 518).

	Univariate analysis for survival		Multivariate analysis for survival	
	Hazard ratio	*P* value	Hazard ratio	*P* value
Statin use				
No	Reference		Reference	
Yes	0.47 (0.20–0.93)	**0**.**03**	0.29 (0.09–0.71)	**0**.**004**
Age				
≤57	Reference		Not done	
>57	0.85 (0.60–1.19)	0.34		
Comorbidity				
Medium	Reference		Reference	
High‐very high	1.62 (1.12–2.31)	**0.01**	1.67 (1.07–2.54)	**0.02**
Performance status				
<70	Reference		Not done	
≥70	0.62 (0.32–1.38)	0.22		
Alcohol use				
<3 drinks/day	Reference		Reference	
≥3 drinks/day	1.72 (1.06–2.95)	**0.003**	1.60 (0.98–2.74)	0.06
Smoking				
<10 pack‐year	Reference			
≥10 pack‐year	1.16 (0.76–1.83)	0.50	Not done	
T‐stage				
T1–2	Reference		Reference	
T3–4	1.75 (1.14–2.79)	**0.009**	2.12 (1.23–4.00)	**0.006**
N‐stage				
N0–1	Reference		Reference	
N2–3	1.41 (1.00–2.00)	0.05	1.26 (0.84–1.91)	0.25
Induction chemotherapy				
No	Reference		Not done	
Yes	1.23 (0.86–1.74)	0.24		
Concurrent chemotherapy				
No	Reference		Not done	
Yes	1.21 (0.84–1.80)	0.30		
Post‐operative radiotherapy				
No	Reference		Reference	
Yes	0.52 (0.35–0.75)	**0.0005**	0.53 (0.32–0.84)	**0.006**

For univariate and multivariate analyses (Table 2), Cox proportional hazard models was used to compare differences in survival. Variables with P value < 0.1 on univariate analysis were included on multivariate analysis. Significance was assessed using the Log Rank test. Use of statins, high ‐ very high comorbidity, the size of the tumor and the use of post‐operative radiotherapy have an impact on survival.

**Figure 6:**
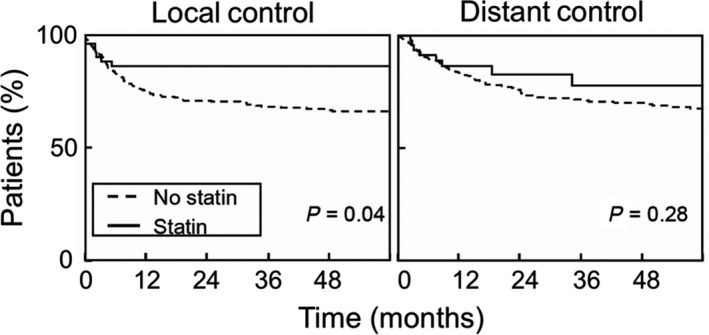
Statin use improves outcomes of radiotherapy in HNSCC patients. Analysis was conducted using data from 518 medical records of stage III‐IVB HNSCC patients, a fraction of whom were incidentally being treated with statins concomitantly with their radiotherapy. Time to local control or distant control was determined from last date of radiotherapy. Survival curves were plotted using the Kaplan–Meier method, and significance was assessed using the Log Rank test. Data indicate that incidental use of statins was statistically associated with better local, but not distant, control in HNSCC patients.

For univariate and multivariate analyses, Cox proportional hazard models was used to compare differences in survival. Variables with *P* value < 0.1 on univariate analysis were included on multivariate analysis. Significance was assessed using the Log Rank test. Use of statins, high ‐ very high comorbidity, the size of the tumor and the use of post‐operative radiotherapy have an impact on survival. The effect of statins on local and distant control by radiotherapy treatment of HNSCC tumors is shown in Fig. 6. A significant effect on local control of tumors was found for patients taking statins (*P* = 0.04), with ≥ 90% of patients on a statin drug achieving local control for up to 48 months. Rates of local control for patients not taking a statin fell to 70% by 12 months post‐treatment. Unfortunately, no significant effect was observed for distant control of HNSCC tumor metastasis (*P* = 0.28), implying that statin drugs are most effective in local rather than distant tumor control.

## 4 Discussion

Although many head and neck squamous cell cancers (HNSCC) initially regress upon treatment with conventional or targeted therapies and display a complete or partial response, a large fraction recur locally or at distant sites with metastatic disease. For recurrent HNSCC, the prognosis remains poor as these tumors typically display acquired therapy resistance. This pattern likely reflects the heterogeneity of epithelial tumors and treatment driving selection for cancer cells able to survive and proliferate even in the presence of therapy. When local recurrence occurs after radiotherapy, this can be particularly problematic, given the limited tolerance of normal tissue for reirradiation. Were it possible to prevent the selection of resistant clones and thus suppress the emergence of resistant disease, recurrence might be rarer and easier to treat. In the case of locoregional recurrence, sensitizers targeting resistance might permit reirradiation at tolerable doses.

This work identifies the mevalonate pathway as a promising target to overcome radiation resistance in HNSCC. Quantitative proteomic profiling to compare two radioresistant HNSCC cell lines, SQ20B and JSQ3, to the radiosensitive cell line SCC61 revealed altered expression of several mevalonate pathway enzymes. Further analysis revealed increased expression of HMG‐CoA reductase (HMGCR), the rate‐limiting enzyme of mevalonate synthesis, in the radioresistant cells. In turn, the radioresistant cells also displayed increased cholesterol levels. Treating SQ20B and JSQ3 with PIT only partly suppressed cholesterol levels, suggesting active uptake of cholesterol along with increased biosynthesis. Import of free cholesterol was similar between the three cell lines, but SQ20B and JSQ3 displayed higher expression of LDL receptors and greater LDL uptake compared with SCC61. Despite the moderate impact on cellular cholesterol, PIT and other lipophilic statins decreased cell proliferation and viability in the radioresistant HNSCC cells. Further, when combined with IR, PIT restored radiation sensitivity to SQ20B and JSQ3 cells, increasing cell death, DNA damage persistence, and senescence. These latter effects may be partly independent of cholesterol levels, potentially mediated by other metabolic pathways downstream of HMGCR and mevalonate biosynthesis such as protein prenylation as previously observed (Efimova *et al*., 2017). Even so, the active uptake of cholesterol by the radioresistant cell lines SQ20B and JSQ3 reinforces the impression that high cellular cholesterol may be critical for their survival and proliferation.

Prior studies have shown that the mevalonate pathway in cancer cells can impact cell division, tumor growth, and metastatic potential (Clendening *et al*., 2010) (Thurnher *et al*., 2012) and modulate immune response and mTOR signaling (Bekkering *et al*., 2018). Recently, novel links between the mevalonate pathway and apoptosis, cell cycle arrest, and DNA damage response have been observed (Horlbeck *et al*., 2018; Martin Sanchez *et al*., 2015; Stine *et al*., 2016). Our prior work demonstrated strong radiosensitization by statin drugs in breast cancer and melanoma cell lines both *in vitro* and *in vivo* (Efimova *et al*., 2017). While connections have been noted between the mevalonate pathway and tumor radioresistance in prostate and pancreatic cancer (Chen *et al*., 2018; Souchek *et al*., 2014), this study is the first to extend these links to HNSCC.

Given the strong influence of advanced age and smoking history on head and neck cancer, it is not surprising that a large fraction of patients also display intercurrent cardiovascular disease and/or hypercholesterolemia, leading to high rates of incidental statin use. In our analysis, among HNSCC patients treated with radiotherapy, taking statins appeared to improve locoregional control, suggesting radiosensitization *in vivo*. Additional studies would be useful to reduce potentially confounding variables such as HNSCC subtype, HPV status, and patient age. Expanding the analysis to include more patients taking statins would allow analysis of the potential impact of specific types of statin, if any. However, our data mining approach utilizing currently available patient data revealed promising findings regarding local control of tumors. We hope these findings will help to justify future clinical studies involving co‐treatment with lipophilic statins for HNSCC patients undergoing radiotherapy.

## 5 Conclusions

In radioresistant HNSCC cell lines, we observed dysregulation of proteins in the mevalonate pathway, as well as elevated cholesterol content, increased LDL receptor abundance, and high levels of LDL uptake. Radioresistant cells displayed loss of cell viability, decreased clonogenic potential, an increase in DNA damage, and induction of cell senescence when treated with the mevalonate pathway inhibitor pitavastatin along with radiation. As a clinical correlate, head and neck cancer patients taking statins displayed improved local tumor control after radiation therapy, suggesting value for administering lipophilic statins to HNSCC patients as a radiosensitization strategy.

## Conflict of interest

S.J.K. is a founder of OncoSenescence. The other authors have no conflicts to declare.

## Author contributions

NR, EVE, AWT, and SJK involved in the conception and design. NR, DW, AF, EVE, and SJK involved in the development of methodology. NR, DW, AF, EVE, AR, OKA, JB, and MTS involved in the acquisition of data. NR, DW, AF, EVE, AR, OKA, MTS, and SJK analyzed and interpreted the data. NR, AF, DW, EVE, AR, OKA, JB, AWT, MTS, and SJK wrote, reviewed, and/or revised the manuscript.

## Supporting information


**Fig. S1.** Metabolism‐related Gene Ontology (GO) analysis of radioresistant HNSCC proteomes. Proteins were separated into functional categories and represented as the log_2_ ratio for JSQ3/SCC61 (left) and SQ20B/SCC61 (right). Significantly differentiated metabolism‐related GO categories found in both radioresistant cell lines include glucose metabolic process, protein metabolic process, and lipid metabolic process. *P* values for each GO category were determined using the Panther GO database and are shown in the figure.

**Fig. S2.** Downregulation of critical mevalonate pathway enzymes HMGCS1 and NSDHL. Cells were harvested in active growth phase, lysed, and subjected to Western blotting for targets as shown. Actin is shown as a protein loading control. HMGCS1 and NSDHL were both downregulated in radioresistant cell lines JSQ3 and SQ20B, consistent with proteomics data.

**Fig. S3.** Combination treatment of pitavastatin and irradiation induces persistent DNA damage and accelerated senescence in JSQ3 radioresistant cells. (A) Plots representing mean percent of DNA in comet tail ± SEM for 100 cells per treatment condition. Significance is indicated by *, *P* < 0.05 (Mann–Whitney U‐test). Results indicate that PIT alone enhances persistent DSBs in radioresistant JSQ3 cells. PIT + IR had a similar effect. (B) Flow cytometric senescence assay data of cells treated with 10 μm PIT (PIT), 10 Gy (IR), or PIT + IR. Vehicle‐treated cells (VEH) were stained as controls. PIT + IR significantly induced senescence in the radioresistant cells above IR or PIT only treatments. (C) Statistical significance was determined using Mann–Whitney U‐test. Mean fluorescent intensity (MFI) of the senescence probe is shown. Error bars, SEM. *, *P* < 0.05; ns, not significant. *n* > 3000 viable cells per sample.

**Fig. S4.** Colony formation assay of SCC61, JSQ3, and SQ20B treated with statin ± IR. Cells were treated with 2.5, 5 or 10 μm PIT, simvastatin, lovastatin, atorvastatin, pravastatin or rosuvastatin for 1 h prior to 10 Gy irradiation. After 4 d of culture, crystal violet staining was conducted and plates were imaged.

## Data Availability

Research data pertaining to this article is located at figshare.com: https://doi.org/10.6084/m9.figshare.8145971.
