# Supplementary figures and images for: Mevalonate pathway activity as a determinant of radiation sensitivity in head and neck cancer
Source: Mol Oncol. 2019 Jul 26;13(9):1927–43. doi: 10.1002/1878-0261.12535 (PMC6717759; doi:10.1002/1878-0261.12535)

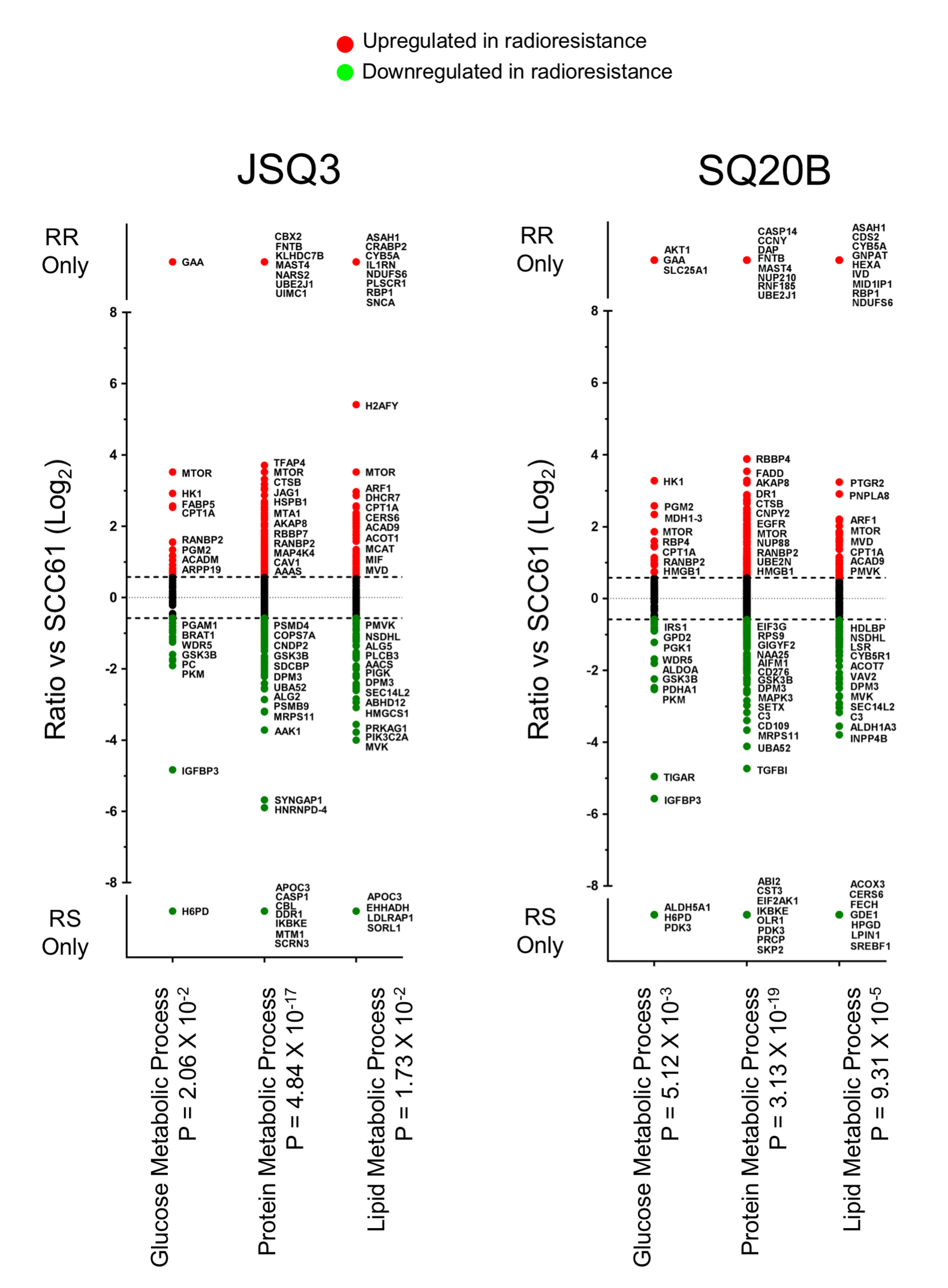

Supplement: Supplementary file 1 — Fig. S1. Metabolism‐related Gene Ontology (GO) analysis of radioresistant HNSCC proteomes. Proteins were separated into functional categories and represented as the log2 ratio for JSQ3/SCC61 (left) and SQ20B/SCC61 (right). Significantly differentiated metabolism‐related GO categories found in both radioresistant cell lines include glucose metabolic process, protein metabolic process, and lipid metabolic process. P values for each GO category were determined using the Panther GO database and are shown in the figure. [file MOL2-13-1927-s001.tif]

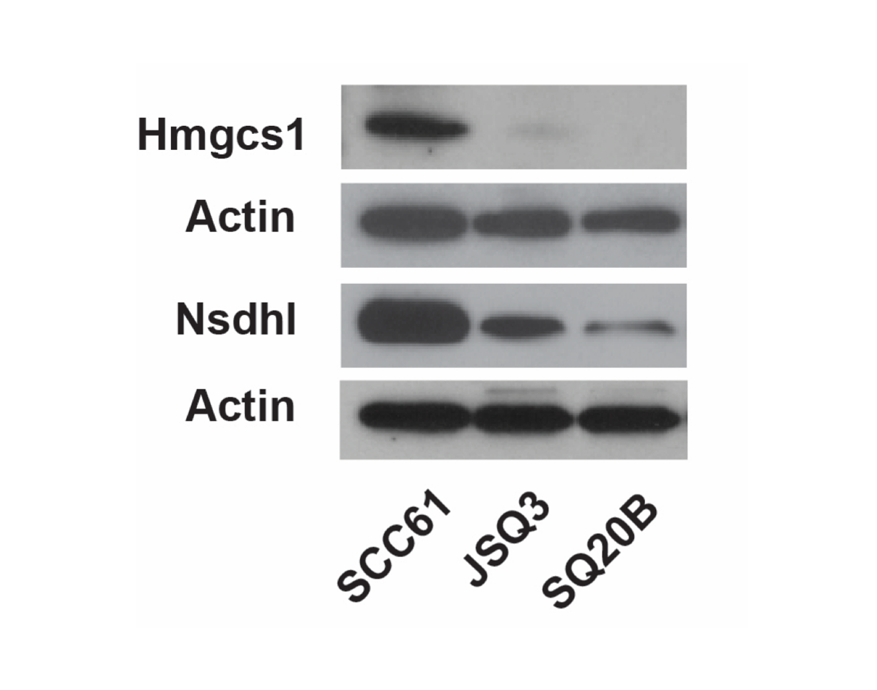

Supplement: Supplementary file 2 — Fig. S2. Downregulation of critical mevalonate pathway enzymes HMGCS1 and NSDHL. Cells were harvested in active growth phase, lysed, and subjected to Western blotting for targets as shown. Actin is shown as a protein loading control. HMGCS1 and NSDHL were both downregulated in radioresistant cell lines JSQ3 and SQ20B, consistent with proteomics data. [file MOL2-13-1927-s002.tif]

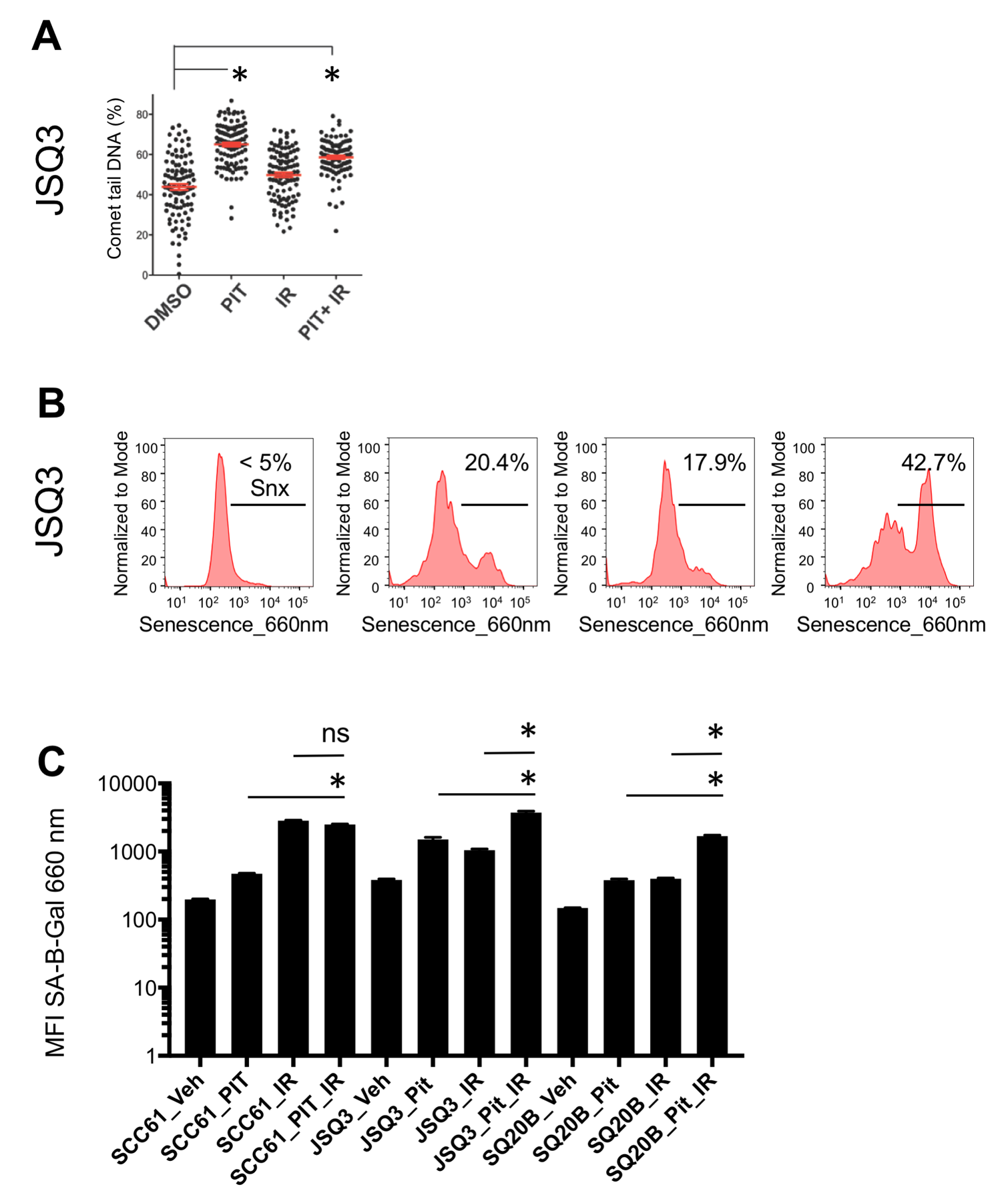

Supplement: Supplementary file 3 — Fig. S3. Combination treatment of pitavastatin and irradiation induces persistent DNA damage and accelerated senescence in JSQ3 radioresistant cells. (A) Plots representing mean percent of DNA in comet tail ± SEM for 100 cells per treatment condition. Significance is indicated by *, P < 0.05 (Mann–Whitney U‐test). Results indicate that PIT alone enhances persistent DSBs in radioresistant JSQ3 cells. PIT + IR had a similar effect. (B) Flow cytometric senescence assay data of cells treated with 10 μm PIT (PIT), 10 Gy (IR), or PIT + IR. Vehicle‐treated cells (VEH) were stained as controls. PIT + IR significantly induced senescence in the radioresistant cells above IR or PIT only treatments. (C) Statistical significance was determined using Mann–Whitney U‐test. Mean fluorescent intensity (MFI) of the senescence probe is shown. Error bars, SEM. *, P < 0.05; ns, not significant. n > 3000 viable cells per sample. [file MOL2-13-1927-s003.tif]

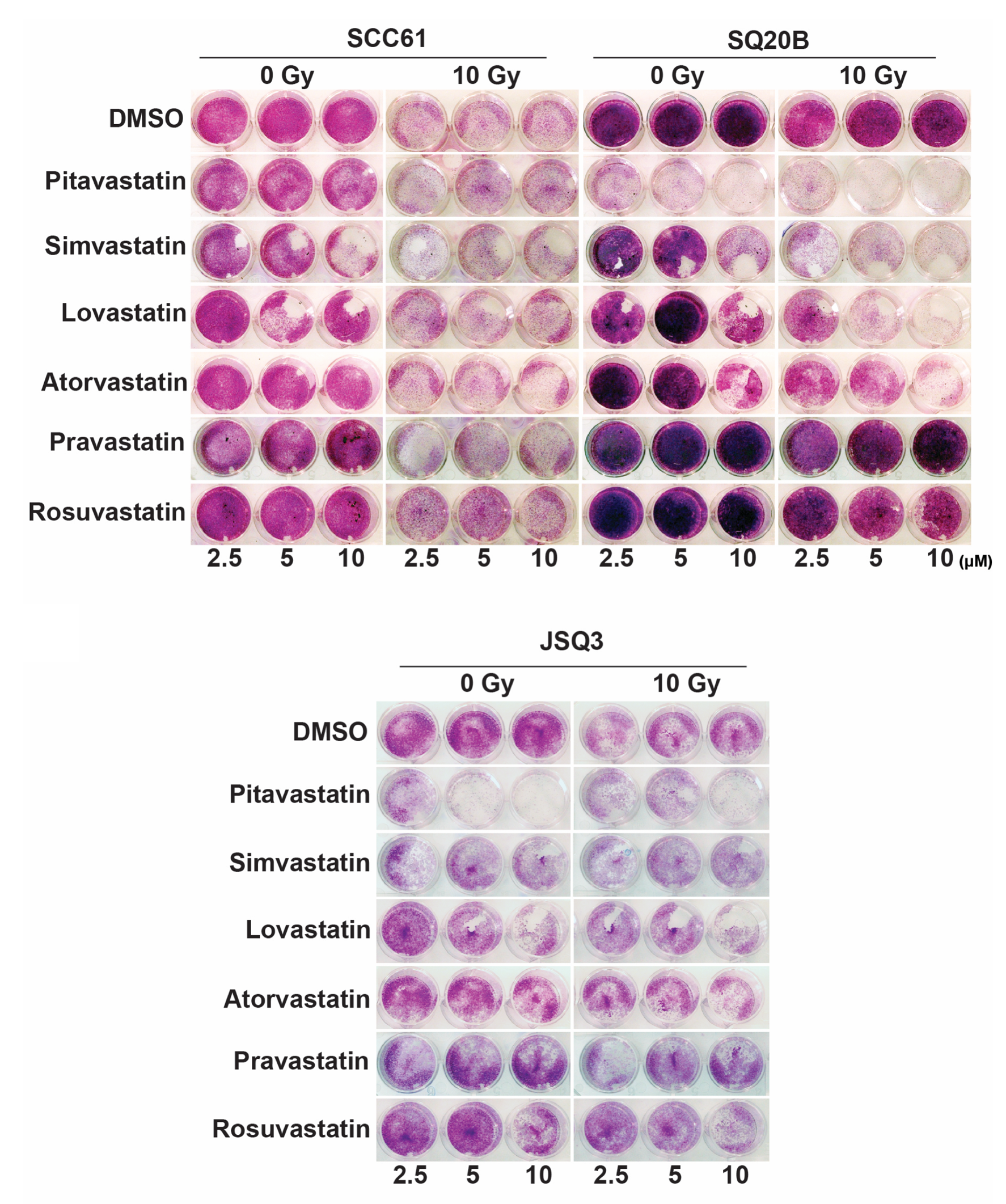

Supplement: Supplementary file 4 — Fig. S4. Colony formation assay of SCC61, JSQ3, and SQ20B treated with statin ± IR. Cells were treated with 2.5, 5 or 10 μm PIT, simvastatin, lovastatin, atorvastatin, pravastatin or rosuvastatin for 1 h prior to 10 Gy irradiation. After 4 d of culture, crystal violet staining was conducted and plates were imaged. [file MOL2-13-1927-s004.tif]
